# Enhanced recovery after surgery protocols in total knee arthroplasty via midvastus approach: a randomized controlled trial

**DOI:** 10.1186/s12891-021-04731-6

**Published:** 2021-10-08

**Authors:** Bo Wei, Cheng Tang, Xuxiang Li, Rongcai Lin, Liu Han, Suyang Zheng, Yan Xu, Qingqiang Yao, Liming Wang

**Affiliations:** 1 Department of Orthopaedic Surgery, Nanjing First Hospital, Nanjing Medical University, Nanjing, 210006 Jiangsu China; 2 Digital Medicine Institute, Nanjing Medical University, Nanjing, 210006 Jiangsu China; 3 Department of Anesthesiology, Nanjing First Hospital, Nanjing Medical University, Nanjing, 210006 Jiangsu China

**Keywords:** Midvastus, ERAS, Optimization management, TKA

## Abstract

**Background:**

Enhanced recovery after surgery (ERAS) protocols were rapidly adopted in many surgeries such as fast-track arthroplasty. The study aimed to investigate the impact of ERAS protocols on the clinical effect of total knee arthroplasty (TKA) via the midvastus approach.

**Methods:**

A total of 69 patients who underwent primary unilateral TKA via the midvastus approach from October 2018 to June 2019 were enrolled and randomly divided into two groups: ERAS group and Control group. The ERAS protocols were adopted for the ERAS group and consisted of pure juice drinking 2 h before the surgery, optimization of the preoperative anesthesia plan, phased use of tourniquets, and the use of tranexamic acid as well as a drug cocktail. The operative time, first postoperative walking time, first straight leg elevation time, postoperative hospitalization time, visual analogue scale score (VAS score), Hospital for Special Surgery score (HSS score), conventional Knee Society score (KSS), and knee range of motion (ROM) were used to assess the clinical effects in the two groups. All the included patients were followed up for 12 months.

**Results:**

There were no significant differences in the basic demographic information and operation time between the ERAS and Control groups (*P* > 0.05). The first postoperative walking time (2.11 ± 0.11 h) and first postoperative straight leg elevation time (6.14 ± 1.73 h) in the ERAS group were significantly earlier than those in the Control group (*P* < 0.001) and the postoperative hospitalization time was significantly shorter (3.11 ± 0.32 days). The postoperative mean VAS scores in both groups were significantly reduced compared with those before surgery (*P* < 0.001). The VAS scores for the ERAS group were significantly lower than those for the Control group at 1, 2, and 7 days after surgery (P < 0.001). The mean HSS scores, KSS, and knee ROM were significantly increased in both the ERAS and Control groups at 1, 3, 6, and 12 months after surgery (*P* < 0.001). In addition, the HSS scores, KSS, and knee ROM in the ERAS group were significantly higher than those in the Control group at 1 month after surgery (*P* < 0.001).

**Conclusions:**

ERAS protocols improved the clinical effects of TKA via the midvastus approach, facilitating early out-of-bed activity and comfortable postoperative rehabilitation exercise, and further increasing patient satisfaction.

**Trial registration:**

ClinicalTrials.gov Identifier: NCT04873544.

## Background

Total knee arthroplasty (TKA) is an effective treatment for end-stage knee osteoarthritis, as it can significantly alleviate pain symptoms and improve knee function [1]. Knee functional activities may be limited to a certain extent after TKA, especially when a kneeling or squatting position is involved [2]. The reported dissatisfaction rate after knee replacement is as high as 19%, with 14–28% of dissatisfaction attributed to pain relief and 16–30% to knee function recovery [3]. Patients with more surgical satisfaction generally indicated that their knee pain and function improved after surgery, while those with less surgical satisfaction indicated that their symptoms and functional limitations were not completely resolved [4]. However, residual pain and persistent dysfunction after TKA are not uncommon [5]. Conventionally, TKA with the medial parapatellar approach can damage the quadriceps femoris tendon. The weakening of the extensor device may cause quadriceps femoris muscle weakness and femoral pain after surgery, which will in turn limit early rehabilitation exercises. Chareancholvanich et al. [6] found that the length of the quadriceps incision could impact the postoperative muscle strength, postoperative pain, and swelling of the knee joint. Specifically, if the quadriceps incision length was controlled within 4 cm, the postoperative recovery of the quadriceps strength was not significantly affected during the perioperative period.

In 1997, Kehlet et al. [7] first proposed the concept of enhanced recovery after surgery (ERAS). ERAS protocols were rapidly adopted in many surgeries such as fast-track arthroplasty with the goal of reducing surgical trauma and the length of hospital stay and hastening postoperative recovery [8, 9]. Minimally invasive approaches for TKA include the midvastus approach, medial subvastus approach, and lateral subvastus approach [10]. Each TKA approach had different advantages and disadvantages, and the ERAS protocols have been confirmed to promote early recovery from lower extremity joint arthroplasty [11]. In this study, a randomized controlled study was conducted to investigate whether our ERAS protocols in TKA via the midvastus approach would affect: (1) the postoperative hospitalization time; (2) the operative time, first postoperative walking time and first straight leg elevation time; (3) visual analogue scale score (VAS score), Hospital for Special Surgery score (HSS score), conventional Knee Society score (KSS) and knee range of motion (ROM).

## Methods

### Inclusion and exclusion criteria

This study adheres to CONSORT guideline and was registered in the ClinicalTrials.gov (registration number: NCT04873544). The clinical trial was approved by the local ethics board (ethics number: KY20170418–02), and written informed consent was obtained prior to the enrollment. All the methods were conducted according to the CONSORT 2010 statement. The inclusion criteria for this study were as follows: 1) unilateral primary knee osteoarthritis; 2) varus, valgus, and flexion contracture deformity of the knee joint < 10°; 3) the ROM of the knee joint was > 80°; 4) body mass index (BMI) < 30; 5) no previous history of knee surgery on the affected side; 6) agreed to participate in the trial. The exclusion criteria for this research were as follows: 1) rheumatoid arthritis; 2) extra-knee deformity; 3) simultaneous bilateral knee arthroplasty; 4) severe dysfunction of the liver, kidney, and blood system; 5) severe cardiovascular diseases; 6) gastrointestinal ulcer; 7) other medical conditions like nausea, vomiting, snoring, and allergy to related analgesic drugs.

### Grouping, randomization and outcomes

A total of 84 patients from the outpatient department were included in this trial. The recruited patients were randomly divided into two groups using sealed envelopes opened before surgery, namely the ERAS group and control group. All TKA surgeries were performed by the same team of surgeons. All patients received an Evolution® medial pivot prosthesis from MicroPort, Inc. (Arlington, TN). All patients were treated with the midvastus approach. The researchers who performed randomization and outcome measurements were different from the surgeons who conducted the TKA. The primary outcome was the postoperative hospitalization time and the secondary outcomes were the operative time, first postoperative walking time, first straight leg elevation time, VAS score, HSS score, conventional KSS and knee ROM.

### Preoperative preparation and evaluation

Preoperative anteroposterior and lateral X-ray films of the targeted knee joints, full-length X-ray films, and 3D computed tomography (CT) of both lower extremities were conducted for both groups. Thus, we could accurately measure the alignment of the lower limbs before TKA, which was beneficial for the selection of the osteotomy angle during the surgery. According to the guidelines from the European Society for Clinical Nutrition and Metabolism in 2017 [12], patients in the ERAS group could drink 200 ml of slag-free 100% pure fruit juice (Weiquan daily C series juice, containing 12.4 g of carbohydrate per 100 ml) 2 h before the operation. The carbohydrate content of the drink in the ERAS group was similar to that of the commonly used preOp® drink from the Nutricia company in the Netherlands [13], and the gastric residue was further evaluated by ultrasound before anesthesia. The contraindications for preoperative oral beverages were reflux esophagitis, gastric motility disorder, special gastrointestinal surgery history, previous or current symptoms of digestive tract obstruction, and diabetic patients with poor preoperative blood glucose control. Patients in the ERAS group were anesthetized with ultrasound-guided adductor canal block (blocking the saphenous nerve), combined with the popliteal artery and capsule of the knee (IPACK) block (blocking the branches of the tibial nerve, common peroneal nerve, and obturator nerve). General anesthesia with a laryngeal mask airway was also adopted during the operation to achieve sufficient analgesia, appropriate sedation, and full muscle relaxation. In the Control group, preoperative fasting and abstinence from drinking were routine, and general anesthesia with a laryngeal mask airway was adopted.

### Surgical procedure

Leg lifting was used to drive the blood during the operation in both groups. In the ERAS group, a tourniquet was applied periodically (no tourniquet was used from the skin incision to osteotomy, and a tourniquet was used from the installation of the prosthesis to wound suture and bandage). In the Control group, a tourniquet was used during the entire surgical process after 30 s of leg lifting to expel blood. None of the patients received a patellar replacement, and no drainage tube and urethral catheter were placed after the operation. The skin was sutured intradermally without removing the stitches. The patients in both groups received 1 g tranexamic acid by intravenous drip 30 min prior to the operation and 0.5 g tranexamic acid by local irrigation. All TKA surgeries were performed by the same team of surgeons. All patients received an Evolution® medial pivot prosthesis from MicroPort, Inc. (Arlington, TN).

All included patients were treated with the midvastus minimally invasive approach as follows: an anterior median incision (starting from the upper edge of the patella and ending on the medial side of the tibial tubercle, the length of the incision was about 10–11 cm) was made in the targeted knee. After exposure, an oblique vastus medialis incision 1–2 cm in length was made 30 degrees along the superior pole and longitudinal axis of the patella. Then, the joint cavity was exposed through the medial parapatellar incision. The quadriceps femoris and patella were pushed to the outside without turning over the patella.

**Fig. 1:**
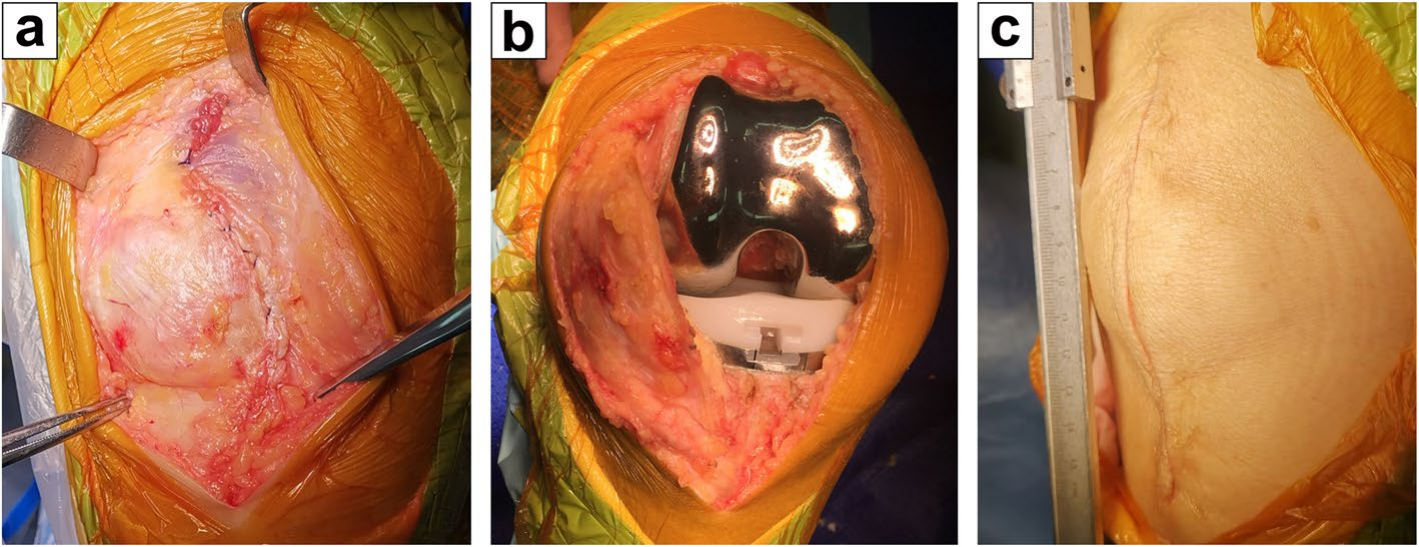
The intraoperative photographs of TKA via the midvastus approach. **a** The oblique vastus medialis and medial parapatellar incision. **b** The medial pivot knee prosthesis. **c** The skin incision of 10–11 cm in length was sutured intradermally without drainage tubes

During the operation, the “moving window” technique was used to expose the operated area in turn, and the gap balance technique was used to complete the osteotomy and soft tissue balance. After confirming the balance of knee flexion and extension, the knee prosthesis was installed using cement fixation. Then, the patella surface was formed and peripheral denervation was performed. The prepared “cocktail” (0.75% ropivacaine 20 ml (150 mg), dexamethasone 1 ml (5 mg), and normal saline added up to 60 ml) was injected into the joint capsule, extensor device, patellar ligament, muscle, and subcutaneous tissue around the knee joint. The incision was sutured layer by layer without drainage tubes, and the skin was sutured intradermally and bandaged with elastic bandage (Fig. 1).

### Postoperative management and evaluation

At the end of the surgery, all patients were connected to an electronic patient-controlled intravenous analgesia pump (PCIA pump) (loading dose: 5 mg oxycodone, single dose: 1 mg oxycodone, locking time: 5 min, background infusion: 0.5 ml/h). Patients in the ERAS group drank pure fruit juice in the postanesthesia care unit (PACU) and began to walk with a walking aid 2 h after the operation. After returning to the ward, the patients were instructed to perform ankle pumps, lower limb straight leg raising exercises, and knee flexion and extension exercises. The time of the first postoperative straight leg raising exercise was evaluated. Intravenous non-steroidal anti-inflammatory analgesics were routinely used. On the first and second days after the operation, functional exercises were conducted under the guidance of rehabilitation physicians, including quadriceps femoris muscle strength exercises and knee flexion and extension exercises. The patients were discharged about 3 days after the operation. They were prescribed oral non-steroidal anti-inflammatory and analgesic drugs and instructed to continue the rehabilitation exercises at home as required. VAS scores were assessed on days 1 and 2 after the operation, as well as 1 week later in the outpatient department. The VAS scores, HSS scores, KSS, and knee ROM were evaluated in the outpatient department at 1, 3, 6, and 12 months after the operation.

The patients in the Control group underwent the same rehabilitation procedures. However, on the first day after the operation, the patients were encouraged to walk with walking aids. On the second day, the patients began functional exercises under the guidance of rehabilitation physicians. The patients were mostly discharged 1 week after the operation and continued oral nonsteroidal anti-inflammatory analgesics and rehabilitation exercises at home under guidance. The VAS scores, HSS scores, KSS, and knee ROM were assessed with the same protocols as in the ERAS group.

### Statistical analysis

SPSS 18.0 software was used for statistical analysis. The chi-square test was used to compare the differences in gender between the ERAS and Control groups. The measurement data were expressed in the form of the mean ± standard deviation. Two independent samples t-tests were used to compare the differences in the age, BMI, operation time, postoperative first walking time, postoperative first straight leg raising time, postoperative hospital stay, VAS scores, HSS scores, KSS, and knee ROM between the ERAS and Control groups. The VAS scores, HSS scores, KSS, and ROM of the knee joint in each group at different times were compared using one-way analysis of variance (ANOVA), and *P* < 0.05 was considered statistically significant.

## Results

### Demographic information

**Fig. 2:**
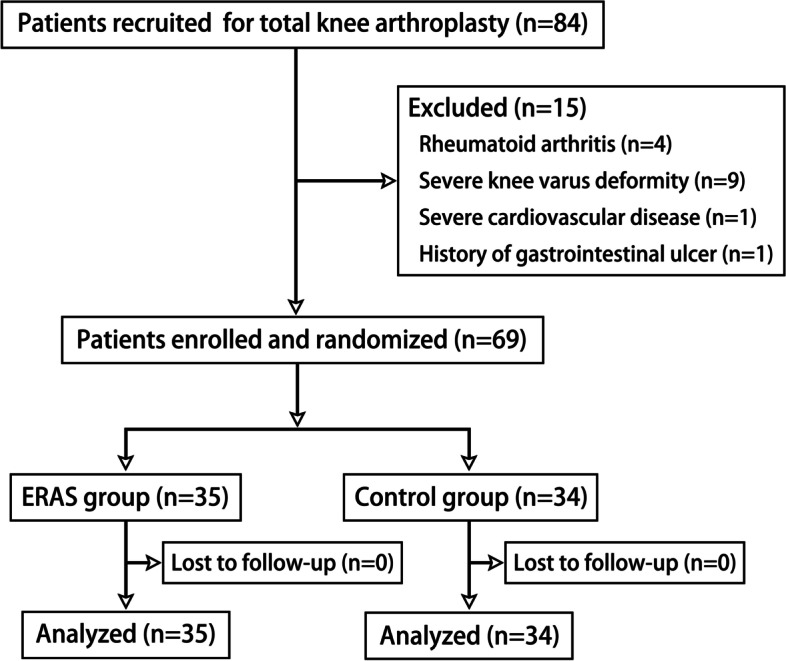
Flow diagram for patient enrollment

A total of 84 patients who underwent initial unilateral TKA in the Department of Orthopaedic Surgery from October 2018 to June 2019 were recruited from the outpatient department. Fifteen patients were excluded, specifically four patients with rheumatoid arthritis, nine with severe knee varus deformity, one with severe cardiovascular disease, and one with a history of gastrointestinal ulcer. A total of 69 patients were randomly divided into the ERAS group and the Control group (Fig. 2). The ERAS group contained 35 patients, including 7 men and 28 women. The Control group contained 34 patients, including 7 men and 27 women. There were no significant differences in age, BMI, preoperative VAS scores, HSS scores, KSS, and knee ROM between the ERAS and Control groups (*P* > 0.05) (Table 1).

**Table 1:** Comparison of the baseline information between the ERAS and Control groups

Group	Age (years)	BMI (kg/m2)	Preoperative VAS scores (points)	Preoperative HSS scores (points)	Preoperative KSS (points)	Preoperative knee range of motion (degrees)
ERAS group	65.77 ± 4.51	26.66 ± 1.19	5.51 ± 0.66	51.23 ± 1.78	46.03 ± 2.05	91.14 ± 3.49
Control group	65.62 ± 5.06	26.91 ± 1.22	5.74 ± 0.71	51.53 ± 2.11	46.09 ± 1.86	91.53 ± 3.18
Statistic	t = 0.133, *P* = 0.894	t = −0.881, *P* = 0.382	t = −1.342, *P* = 0.184	t = −0.641, *P* = 0.524	t = − 0.126, *P* = 0.900	t = − 0.480, *P* = 0.633

### Clinical outcomes

There was no significant difference in the operation time between the two groups (P > 0.05). The intraoperative blood loss in the ERAS group was significantly higher than that in the Control group (negligible). The first postoperative walk time, the first postoperative straight leg elevation time, and the length of postoperative hospital stay in the ERAS group were significantly shorter than those in the Control group (*P* < 0.001) (Table 2).

**Table 2:** Comparison of the clinical outcomes between the ERAS and control groups

Group	Operation time (minutes)	Intraoperative blood loss (ml)	The first postoperative activity time out of bed (hours)	The first postoperative straight leg raising time (hours)	The postoperative hospitalization time (days)
ERAS group	84.66 ± 3.38	185.20 ± 9.70	2.11 ± 0.11	6.14 ± 1.73	3.11 ± 0.32
Control group	83.85 ± 4.14	134.56 ± 13.51	18.91 ± 2.67	16.44 ± 2.97	7.06 ± 0.60
Statistic	t = 0.885, *P* = 0.379	t = 17.93, *P* < 0.001	t = −37.26, *P* < 0.001	t = −17.67, *P* < 0.001	t = −34.14, *P* < 0.001

**Fig. 3:**
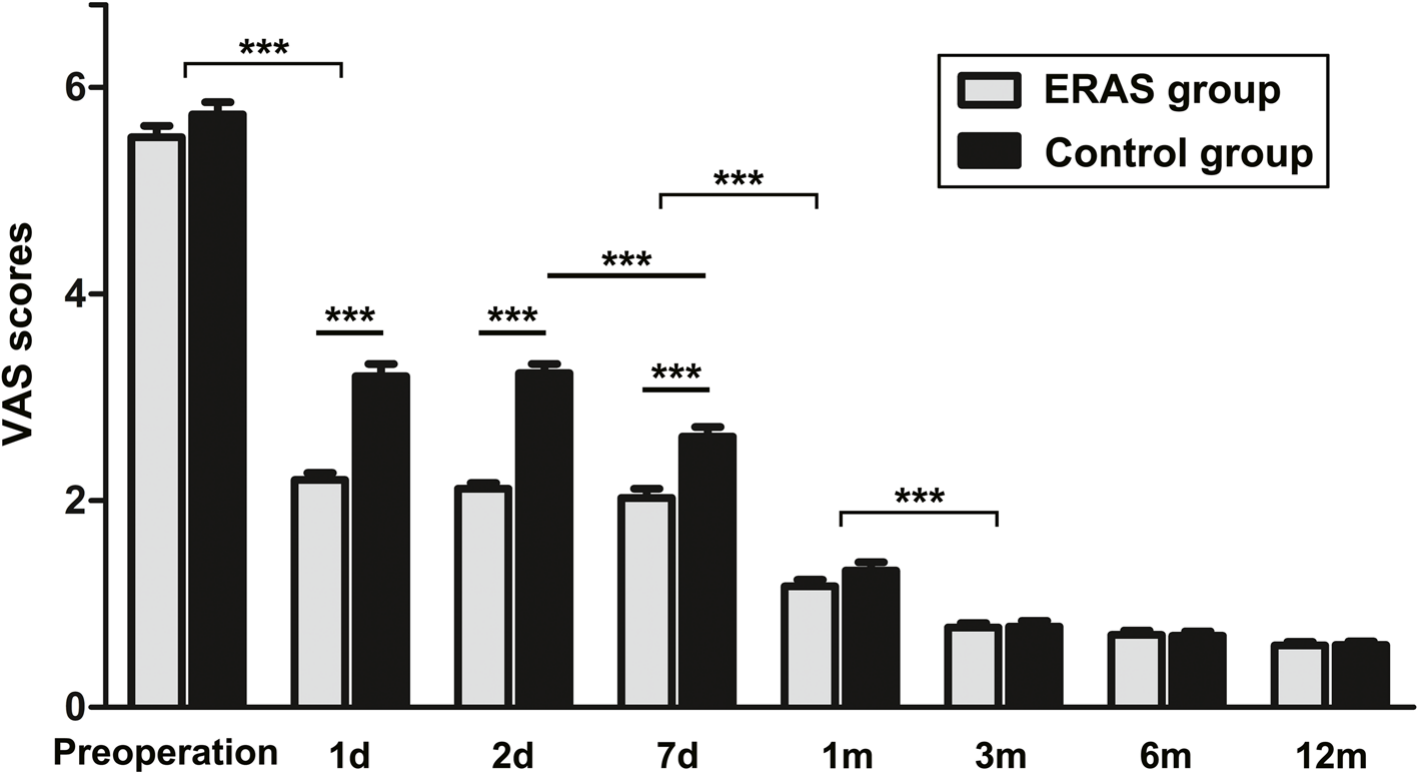
The VAS scores pre- and post-TKA. (*** *P* < 0.001)

The mean postoperative VAS scores in both groups were significantly lower than those before surgery and there were significant differences in the VAS scores measured at 7 days and at 1 and 3 months after the operation (*P* < 0.001). On the 1st, 2nd, and 7th days after the operation, the VAS scores in the ERAS group were significantly lower than those in the Control group (P < 0.001). Additionally, the VAS scores of the ERAS group did not significantly decrease on days 1–7 after the surgery (*P* > 0.05), whereas the VAS scores of the Control group on day 2 after the surgery were significantly higher than those on day 7 (*P* < 0.001). There were no significant differences in the VAS scores between the two groups in the 1-, 3-, 6-, and 12-month follow-up (*P* > 0.05) (Fig. 3).

**Fig. 4:**
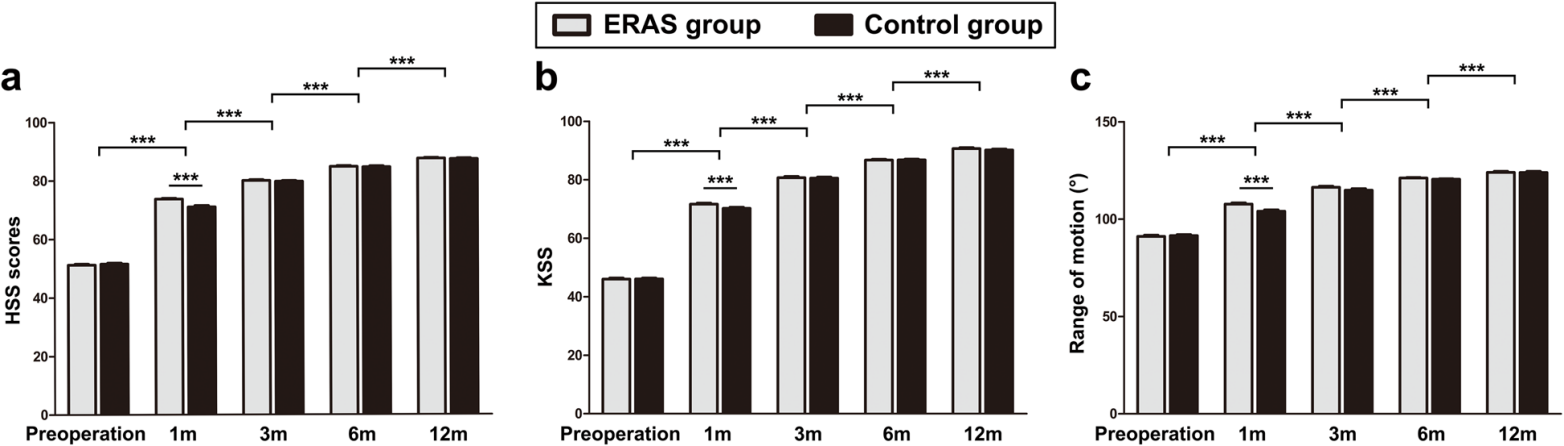
The HSS scores, KSS, and knee ROM pre- and post-TKA. (*** *P* < 0.001)

The average HSS scores, KSS, and knee ROM were significantly increased in both the ERAS and Control groups on the 1st, 3th, 6th, and 12th months after the surgery, and were significantly improved compared with those before the operation (*P* < 0.001). In addition, the HSS scores, KSS, and knee ROM in the ERAS group were significantly higher than those in the Control group in the 1st month (P < 0.001). On the 3th, 6th, and 12th months, no significant differences were observed between the two groups (P > 0.05) (Fig. 4).

## Discussion

Our present study demonstrated that patients following the ERAS protocols could walk with a walking aid about 2 h after surgery. Moreover, the ERAS protocols reduced the first postoperative straight leg elevation time and the average postoperative hospital stay, and improved the early postoperative clinical scores and knee functional activities within 1 month following TKA.

Conventionally, preoperative fasting and drinking abstinence can effectively prevent the occurrence of regurgitation and aspiration in patients undergoing elective surgery, but can cause discomfort such as thirst, hunger, hypoglycemia, tension, and anxiety before and after surgery [14]. With the development of ERAS, the guidelines from the Society for Enhanced Recovery After Surgery [15] and the European Society for Clinical Nutrition and Metabolism [12] both recommend the intake of carbohydrate drinks 2 h before surgery. Carbohydrate supplementation can increase glycogen reserves, improve insulin resistance, keep the body in a state of anabolism, and increase the comfort of the patients before and after surgery. Currently, the most commonly used carbohydrate drink is Nutricia PreOp® [12]. Soop et al. [16] found that drinking 100 ml of Nutricia PreOp® 2 h before the surgery could increase insulin sensitivity by 50% immediately after the surgery. In this study, patients were instructed to drink a dreg-free 100% pure juice drink 2 h before surgery. The drink in this trial had a carbohydrate content similar to that of Nutricia PreOp®, but was cheaper and more easily accessible with satisfactory clinical effects.

The goals of postoperative analgesia after TKA are to effectively control the anterior and posterior pain in the knee joint and to preserve the muscle strength as much as possible, which can facilitate early functional exercise. General anesthesia alone results in a high incidence of postoperative nausea and vomiting due to the large amount of opioids involved. Although peripheral nerve block anesthesia alone has an obvious analgesic effect, achieving satisfactory intraoperative analgesia is difficult with only the nerve due to the rich peripheral nerve innervation around the knee joint [17]. It has been reported that an adductor canal block can effectively control the anterior and medial pain in the knee joint by blocking the saphenous nerve, but the effect is not sufficient for controlling moderate to severe pain after TKA, especially for the control of pain in the posterior of the knee [18, 19]. By blocking the sensory branches behind the knee joint, the IPACK block seemed to be a powerful and lasting method (effective analgesic time of a single IPACK block is 8–12 h) to control the pain behind the knee without affecting the muscle strength below the knee, which was conducive to early postoperative functional exercise [20, 21]. In this study, patients in the ERAS group were treated with an ultrasonic-guided adductor canal block combined with IPACK block to achieve precise anesthesia. Combined with the intraoperative general anesthesia with a laryngeal mask airway, full analgesia, appropriate sedation, and adequate muscle relaxation were achieved.

During TKA, a tourniquet is often used in combination with blood repellent in the lower extremity. Through physical squeezing, extrusion can cause ischemia and hypoxia of the targeted limb, and further release inflammatory factors. Moreover, some hidden blood loss can penetrate into the tissue space through extrusion, resulting in postoperative pain and swelling of the targeted limb, thus affecting early postoperative functional exercise. Lower extremity lifting is a safe and effective method to expel blood and can significantly reduce intraoperative muscle injury, postoperative swelling, and pain compared with extrusion to expel blood [22]. The use of a tourniquet during TKA can clear the visual field clear and facilitate surgery, but can often cause postoperative pain and discomfort in the thigh muscles of the targeted limb [23]. It had been indicated that the non-use of tourniquets during TKA can reduce muscle and soft tissue injury in the targeted limb, relieve postoperative thigh muscle pain, shorten the length of hospital stay, and thus promote the early functional recovery of the operated knee [24]. In this study, the lower limbs were lifted for 30 s to expel blood during the operation and the blood was not extruded using a blood drive belt. The minimally invasive midvastus approach was used to reduce injury to the quadriceps femoris. Patients in the ERAS group were treated with tourniquets periodically to minimize disturbance to the extensor device, reduce the postoperative reaction of the quadriceps femoris, effectively control swelling and pain, and accelerate rehabilitation.

Tranexamic acid, an antifibrinolytic drug, can play a hemostatic role by inhibiting the activation of plasminogens and reducing fibrinolytic activity [25]. Huang et al. [26] found that the intravenous infusion of tranexamic acid combined with local administration during the perioperative period of TKA could effectively reduce the bleeding and blood transfusion rates more than simple intravenous infusion or local application. Moreover, some studies found that tranexamic acid could significantly reduce the amount of blood loss and the risk of blood transfusion during TKA. However, there was no significant difference in the hemostatic effect among different methods of administration (such as oral, local, intravenous, or combined administration), various amounts, and frequency [27, 28]. In this study, 1 g tranexamic acid was injected intravenously 30 min before the operation, combined with local irrigation using 0.5 g tranexamic acid during the operation, which achieved a satisfactory intraoperative hemostasis effect. In addition, the use of “cocktail” therapy during TKA can reduce the use of morphine drugs and achieve the effect of postoperative prophylactic analgesia. The formula for the “cocktail” varied, but the main drug was ropivacaine, which is mainly used for blocking sensory nerves without affecting movement when used in small doses [29]. In this study, we prepared a “cocktail” by mixing 20 ml (150 mg) of 0.75% ropivacaine and 1 ml (5 mg) of dexamethasone with normal saline added up to 60 ml, and injected it into the joint capsule, extensor device, patellar ligament, muscle, and subcutaneous tissue around the knee joint, which resulted in a satisfactory postoperative prophylactic analgesia effect.

Postoperative urinary retention is one of the most common complications in the perioperative period for TKA, and urinary catheter indwelling may be required if urinary retention occurs. As an invasive operation, an indwelling urethral catheter may damage the urethral mucosa and cause urinary tract infection, increasing the risk of periprosthetic infection after TKA [30]. In addition, the physical and psychological discomfort caused by indwelling catheters would delay walking and early rehabilitation exercise, as well as the time of hospital discharge [31]. Studies have demonstrated that the postoperative hospital stay and incidence of postoperative urinary tract infection are significantly lower in patients without indwelling catheters [32, 33]. In the current study, patients following the ERAS protocols could drink carbohydrate drinks 2 h before surgery, walk with a walking aid about 2 h after surgery, and eat food 2–4 h after surgery, which greatly reduced the fluid demand during the perioperative period and the risk of postoperative urinary retention.

The placement of drainage following TKA also affects early walking and functional exercise by patients. Studies found that without indwelling drainage after TKA, the amount of postoperative blood loss, the time the patients got out of bed, and the length of hospital stay were significantly reduced [34, 35]. In this study, all potential bleeding sites were carefully and appropriately handled before closing the incision and drainage tubes were not placed. After surgery, the targeted limbs were wrapped with elastic bandage; hence, bleeding from some small blood vessels could be controlled depending on the pressure in the joint cavity. Although the intraoperative blood loss in the ERAS group was significantly higher than that in the Control group (negligible), the blood loss in the ERAS group was acceptable in clinical practice. The use of an electronic PCIA pump after surgery can make postoperative functional exercise more comfortable, coupled with the contributions from tranexamic acid and the drug “cocktail” during the operation, the use of intradermal sutures with no need for suture removal, no catheter placement, and no drainage tube. Patients in the ERAS group drank pure fruit juice at the PACU about 2 h after the operation and started walking at about 2 h postoperatively. The first postoperative straight leg elevation time and the average postoperative hospital stay in the ERAS group were significantly shorter than those in the Control group. Moreover, the VAS scores for the ERAS group were significantly lower than those for the Control group on days 1, 2, and 7, postoperatively. The HSS scores, KSS, and knee ROM in the ERAS group were significantly better than those in the Control group 1 month postoperatively, indicating satisfactory early clinical efficacy.

However, there were some limitations in this study. First, due to the sample size was small, the ERAS protocols improved the short-term postoperative clinical results within 1 month, so the larger studies with a multi-center design are needed in the future. Secondly, the clinical effects of ERAS in this study were the cumulative results of several optimization schemes, but the specific effects of each scheme were not investigated. Thirdly, the study was not a double-blinded trial, as the surgeons and patients were not blinded to the specific intervention. However, the researchers who assessed the clinical outcomes and analyzed the data were completely independent.

## Conclusions

ERAS improved the post-surgical outcomes after TKA via the midvastus approach, including early postoperative knee joint function and activity. The patients in the ERAS group could start walking earlier and safely, and performed early functional exercise comfortably after TKA, resulting in enhanced satisfaction.

## Data Availability

All the data and material are saved in an anonymized repository file folder and available from the corresponding author on reasonable request.

## Abbreviations

ERAS: Enhanced recovery after surgery
TKA: Total knee arthroplasty
VAS score: Visual analogue scale score
HSS score: Hospital for Special Surgery score
KSS: Knee Society score
BMI: Body mass index
CT: Computed tomography
IPACK: The popliteal artery and capsule of the knee
PCIA: Patient-controlled intravenous analgesia
PACU: Postanesthesia care unit
